# 2,3,4,6-Tetra-*O*-acetyl-2-phthalimido-β-d-glucopyran­oside

**DOI:** 10.1107/S1600536810048099

**Published:** 2010-11-27

**Authors:** Giuliana Gervasio, Domenica Marabello, Federica Bertolotti

**Affiliations:** a Dipartimento di Chimica I, F.M. e Centro CrisDi, University of Turin, Via P. Giuria 7, 10125, Torino, Italy

## Abstract

In the crystal structure of the title compound, C_24_H_27_NO_11_, a substituted tetra­acetyl glucopyran­oside derivative, weak inter­molecular C—H⋯O hydrogen bonds link the mol­ecules into ribbons propagated in [010]. The d configuration has been attributed on the basis of the synthesis and the  β anomer has been determined from the structure.

## Related literature

For the synthesis, see: Dahmen *et al.* (1983*a* ▶,*b* ▶, 1984 ▶); Mag­nus­son *et al.* (1981 ▶); Quagliotto *et al.* (2005 ▶). For related structures, see: Ambrosi *et al.* (2002 ▶); Halasz *et al.* (2005 ▶). 
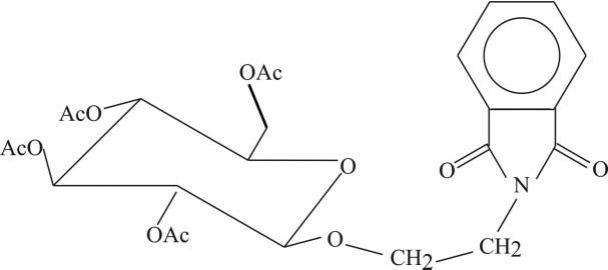


## Experimental

### 

#### Crystal data

C_24_H_27_NO_12_
*M* *_r_* = 521.47
Monoclinic, 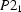

*a* = 10.6447 (8) Å
*b* = 8.3655 (8) Å
*c* = 14.0123 (13) Å
β = 92.263 (2)°
*V* = 1246.80 (19) Å^3^
*Z* = 2
Mo *K*α radiation
μ = 0.11 mm^−1^
*T* = 293 K
0.34 × 0.22 × 0.20 mm

#### Data collection

Bruker APEX diffractometer
Absorption correction: multi-scan (Blessing, 1995 ▶) *T*_min_ = 0.77, *T*_max_ = 1.00
15243 measured reflections
3092 independent reflections
2238 reflections with *I* > 2σ(*I*)
*R*_int_ = 0.036
20 standard reflections every 60 min  intensity decay: none

#### Refinement

*R*[*F* ^2^ > 2σ(*F* ^2^)] = 0.051
*wR*(*F* ^2^) = 0.132
*S* = 1.07
3092 reflections
334 parameters
1 restraint
H-atom parameters constrained
Δρ_max_ = 0.16 e Å^−3^
Δρ_min_ = −0.14 e Å^−3^

### 

Data collection: *SMART* (Bruker, 2007 ▶); cell refinement: *SAINT* (Bruker, 2007 ▶); data reduction: *SAINT*; program(s) used to solve structure: *SHELXS97* (Sheldrick, 2008 ▶); program(s) used to refine structure: *SHELXL97* (Sheldrick, 2008 ▶); molecular graphics: *XP* in *SHELXTL* (Sheldrick, 2008 ▶); software used to prepare material for publication: *SHELXL97*.

## Supplementary Material

Crystal structure: contains datablocks I, global. DOI: 10.1107/S1600536810048099/cv2796sup1.cif

Structure factors: contains datablocks I. DOI: 10.1107/S1600536810048099/cv2796Isup2.hkl

Additional supplementary materials:  crystallographic information; 3D view; checkCIF report

## Figures and Tables

**Table 1:** Hydrogen-bond geometry (Å, °)

*D*—H⋯*A*	*D*—H	H⋯*A*	*D*⋯*A*	*D*—H⋯*A*
C32—H32*B*⋯O42^i^	0.96	2.45	3.317 (5)	151
C43—H43*C*⋯O42^ii^	0.96	2.48	3.284 (5)	141

Symmetry codes: (i) 
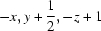
; (ii) 
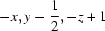
.

**Fig. 1.**
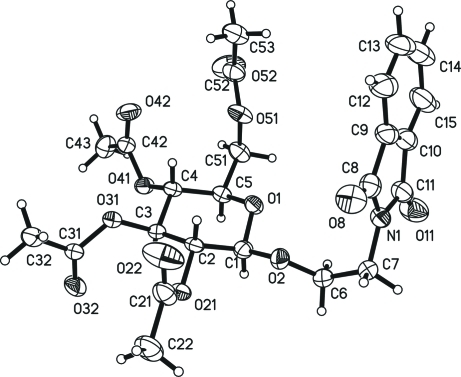
The molecular structure of the title compound showing the atomic numbering and 30% probability displacements ellipsoids.

## supplementary crystallographic information

### Comment

The title compound, 2-phthalimido-2,3,4,6-tetraacetyl-β-D-glucopyranoside, synthesized according to Dahmen *et al.* (1983*a*,*b*; 1984), Magnusson *et al.* (1981) and Quagliotto *et al.* (2005), belongs to the wide category of substituted tetraacetyl D-glucopyranoside compounds and the β anomer has been detected. Bond lengths and angles agree with those observed in numerous similar compounds, for instance, see Ambrosi *et al.* (2002) and Halasz *et al.* (2005). In the crystal structure, weak intermolecular C—H···O hydrogen bonds (Table 1) link the molecules into ribbons propagated in [010] direction.

### Experimental

The title compound has been obtained according to Dahmen *et al.* (1983*a*,*b*;1984) and Magnusson *et al.* (1981).

### Refinement

H atoms have been placed in geometrically idealized positions (C—H = 0.93-0.98 Å), and refined as riding, with *U*_iso_(H) = 1.2-1.5 *U*_eq_(C) The absolute structure cannot be determined reliably from the Flack parameter and, therefore, the 841 Friedel pairs have been merged.

### Crystal data

**Table:**

C_24_H_27_NO_12_	*F*(000) = 548
*M**_r_* = 521.47	*D*_x_ = 1.389 Mg m^−^^3^
Monoclinic, *P*2_1_	Mo *K*α radiation, λ = 0.71073 Å
*a* = 10.6447 (8) Å	Cell parameters from 400 reflections
*b* = 8.3655 (8) Å	θ = 3.0–23.0°
*c* = 14.0123 (13) Å	µ = 0.11 mm^−^^1^
β = 92.263 (2)°	*T* = 293 K
*V* = 1246.80 (19) Å^3^	Prism, colourless
*Z* = 2	0.34 × 0.22 × 0.20 mm

### Data collection

**Table:**

Bruker APEX diffractometer	2238 reflections with *I* > 2σ(*I*)
Radiation source: fine-focus sealed tube	*R*_int_ = 0.036
graphite	θ_max_ = 28.3°, θ_min_ = 1.5°
φ scans	*h* = −14→14
Absorption correction: multi-scan (Blessing, 1995)	*k* = 0→10
*T*_min_ = 0.77, *T*_max_ = 1.00	*l* = 0→18
15243 measured reflections	20 standard reflections every 60 min
3092 independent reflections	intensity decay: none

### Refinement

**Table:**

Refinement on *F*^2^	Primary atom site location: structure-invariant direct methods
Least-squares matrix: full	Secondary atom site location: difference Fourier map
*R*[*F*^2^ > 2σ(*F*^2^)] = 0.051	Hydrogen site location: inferred from neighbouring sites
*wR*(*F*^2^) = 0.132	H-atom parameters constrained
*S* = 1.07	*w* = 1/[σ^2^(*F*_o_^2^) + (0.0605*P*)^2^ + 0.1789*P*] where *P* = (*F*_o_^2^ + 2*F*_c_^2^)/3
3092 reflections	(Δ/σ)_max_ < 0.001
334 parameters	Δρ_max_ = 0.16 e Å^−^^3^
1 restraint	Δρ_min_ = −0.14 e Å^−^^3^

### Special details

**Table:**

Geometry. All e.s.d.'s (except the e.s.d. in the dihedral angle between two l.s. planes) are estimated using the full covariance matrix. The cell e.s.d.'s are taken into account individually in the estimation of e.s.d.'s in distances, angles and torsion angles; correlations between e.s.d.'s in cell parameters are only used when they are defined by crystal symmetry. An approximate (isotropic) treatment of cell e.s.d.'s is used for estimating e.s.d.'s involving l.s. planes.
Refinement. Refinement of *F*^2^ against ALL reflections. The weighted *R*-factor *wR* and goodness of fit *S* are based on *F*^2^, conventional *R*-factors *R* are based on *F*, with *F* set to zero for negative *F*^2^. The threshold expression of *F*^2^ > σ(*F*^2^) is used only for calculating *R*-factors(gt) *etc*. and is not relevant to the choice of reflections for refinement. *R*-factors based on *F*^2^ are statistically about twice as large as those based on *F*, and *R*- factors based on ALL data will be even larger.

### Fractional atomic coordinates and isotropic or equivalent isotropic displacement parameters (Å^2^)

**Table:**

	*x*	*y*	*z*	*U*_iso_*/*U*_eq_	
O1	0.2629 (2)	0.7283 (3)	0.24007 (16)	0.0505 (6)	
C1	0.3802 (3)	0.8111 (5)	0.2432 (3)	0.0491 (9)	
H1A	0.4463	0.7458	0.2745	0.059*	
C2	0.3624 (3)	0.9652 (5)	0.2968 (3)	0.0451 (8)	
H2A	0.2983	1.0301	0.2628	0.054*	
C3	0.3214 (3)	0.9325 (4)	0.3972 (2)	0.0411 (8)	
H3A	0.3905	0.8841	0.4355	0.049*	
C4	0.2076 (3)	0.8243 (4)	0.3953 (2)	0.0440 (8)	
H4A	0.1333	0.8839	0.3722	0.053*	
C5	0.2261 (3)	0.6792 (5)	0.3325 (3)	0.0478 (9)	
H5A	0.2926	0.6121	0.3617	0.057*	
O2	0.4087 (2)	0.8446 (3)	0.14973 (17)	0.0539 (6)	
C6	0.4639 (3)	0.7116 (6)	0.1020 (3)	0.0569 (10)	
H6A	0.4232	0.6130	0.1201	0.068*	
H6B	0.5526	0.7038	0.1201	0.068*	
C7	0.4476 (3)	0.7368 (6)	−0.0042 (3)	0.0567 (10)	
H7A	0.4802	0.8412	−0.0202	0.068*	
H7B	0.4963	0.6571	−0.0368	0.068*	
N1	0.3179 (3)	0.7261 (5)	−0.0376 (2)	0.0558 (8)	
C8	0.2422 (4)	0.8575 (7)	−0.0603 (3)	0.0635 (11)	
O8	0.2759 (4)	0.9941 (5)	−0.0599 (3)	0.0899 (11)	
C9	0.1155 (4)	0.7921 (7)	−0.0853 (3)	0.0689 (13)	
C10	0.1218 (4)	0.6290 (7)	−0.0788 (3)	0.0733 (14)	
C11	0.2527 (4)	0.5832 (7)	−0.0482 (3)	0.0692 (12)	
O11	0.2974 (3)	0.4522 (5)	−0.0343 (3)	0.0964 (12)	
C12	0.0040 (5)	0.8685 (10)	−0.1087 (4)	0.0963 (19)	
H12A	−0.0010	0.9791	−0.1143	0.116*	
C13	−0.1025 (5)	0.7692 (14)	−0.1237 (4)	0.115 (3)	
H13A	−0.1801	0.8165	−0.1376	0.138*	
C14	−0.0955 (6)	0.6067 (15)	−0.1183 (5)	0.120 (3)	
H14A	−0.1676	0.5455	−0.1294	0.144*	
C15	0.0174 (5)	0.5328 (10)	−0.0968 (4)	0.102 (2)	
H15A	0.0236	0.4220	−0.0943	0.122*	
O21	0.4786 (2)	1.0518 (3)	0.30267 (17)	0.0527 (7)	
O22	0.3877 (4)	1.2729 (5)	0.2431 (3)	0.1135 (15)	
C21	0.4783 (4)	1.2067 (6)	0.2740 (3)	0.0664 (12)	
C22	0.6057 (4)	1.2786 (8)	0.2884 (4)	0.0924 (17)	
H22A	0.6036	1.3881	0.2677	0.139*	
H22B	0.6647	1.2201	0.2518	0.139*	
H22C	0.6310	1.2740	0.3549	0.139*	
O31	0.28641 (18)	1.0835 (3)	0.43790 (17)	0.0484 (6)	
O32	0.4534 (2)	1.0863 (4)	0.5412 (2)	0.0775 (9)	
C31	0.3644 (3)	1.1517 (5)	0.5057 (3)	0.0482 (9)	
C32	0.3233 (3)	1.3171 (5)	0.5245 (3)	0.0676 (12)	
H32A	0.3785	1.3645	0.5723	0.101*	
H32B	0.2391	1.3158	0.5466	0.101*	
H32C	0.3255	1.3785	0.4667	0.101*	
O41	0.18882 (19)	0.7666 (3)	0.49050 (17)	0.0511 (6)	
O42	−0.0121 (3)	0.8438 (5)	0.4827 (2)	0.0779 (9)	
C42	0.0715 (3)	0.7777 (5)	0.5246 (3)	0.0519 (9)	
C43	0.0626 (4)	0.6939 (6)	0.6176 (3)	0.0714 (12)	
H43A	−0.0213	0.7039	0.6396	0.107*	
H43B	0.1209	0.7409	0.6635	0.107*	
H43C	0.0825	0.5829	0.6097	0.107*	
O51	0.0126 (2)	0.6793 (4)	0.27174 (19)	0.0653 (8)	
O52	−0.1385 (3)	0.5437 (5)	0.3397 (3)	0.1090 (14)	
C51	0.1083 (3)	0.5800 (6)	0.3180 (3)	0.0626 (11)	
H51A	0.1251	0.4877	0.2786	0.075*	
H51B	0.0801	0.5426	0.3791	0.075*	
C52	−0.1077 (4)	0.6487 (6)	0.2894 (3)	0.0684 (12)	
C53	−0.1945 (4)	0.7581 (8)	0.2382 (4)	0.0872 (15)	
H53D	−0.2794	0.7320	0.2529	0.131*	
H53A	−0.1845	0.7478	0.1707	0.131*	
H53B	−0.1764	0.8660	0.2575	0.131*	

### Atomic displacement parameters (Å^2^)

**Table:**

	*U*^11^	*U*^22^	*U*^33^	*U*^12^	*U*^13^	*U*^23^
O1	0.0478 (12)	0.0564 (15)	0.0467 (13)	−0.0063 (11)	−0.0057 (10)	−0.0029 (13)
C1	0.0432 (17)	0.061 (2)	0.043 (2)	0.0059 (17)	−0.0027 (14)	0.0031 (18)
C2	0.0288 (14)	0.058 (2)	0.048 (2)	−0.0040 (15)	−0.0065 (13)	−0.0030 (17)
C3	0.0302 (14)	0.049 (2)	0.0439 (19)	0.0036 (14)	−0.0037 (13)	−0.0030 (16)
C4	0.0324 (14)	0.052 (2)	0.047 (2)	−0.0034 (14)	−0.0044 (13)	0.0009 (17)
C5	0.0421 (17)	0.051 (2)	0.050 (2)	−0.0020 (16)	−0.0076 (15)	0.0042 (17)
O2	0.0553 (14)	0.0623 (17)	0.0442 (14)	0.0045 (13)	0.0013 (11)	−0.0037 (13)
C6	0.0454 (18)	0.069 (3)	0.056 (2)	0.0075 (18)	−0.0018 (16)	−0.007 (2)
C7	0.0459 (18)	0.071 (3)	0.053 (2)	−0.0039 (19)	0.0065 (16)	−0.010 (2)
N1	0.0499 (16)	0.070 (2)	0.0474 (18)	−0.0045 (17)	−0.0018 (13)	−0.0030 (17)
C8	0.068 (3)	0.077 (3)	0.045 (2)	−0.003 (2)	0.0043 (19)	0.004 (2)
O8	0.091 (2)	0.081 (3)	0.098 (3)	0.000 (2)	−0.001 (2)	0.013 (2)
C9	0.051 (2)	0.109 (4)	0.045 (2)	−0.001 (2)	−0.0030 (17)	0.011 (3)
C10	0.059 (2)	0.106 (4)	0.055 (3)	−0.014 (3)	−0.0029 (19)	0.002 (3)
C11	0.059 (2)	0.087 (4)	0.061 (3)	−0.015 (3)	−0.001 (2)	−0.007 (3)
O11	0.083 (2)	0.076 (3)	0.129 (3)	−0.009 (2)	−0.015 (2)	−0.010 (2)
C12	0.073 (3)	0.151 (6)	0.065 (3)	0.017 (4)	−0.005 (2)	0.021 (4)
C13	0.054 (3)	0.226 (9)	0.063 (3)	0.004 (5)	−0.010 (2)	0.024 (5)
C14	0.071 (4)	0.213 (9)	0.076 (4)	−0.046 (5)	−0.015 (3)	0.022 (5)
C15	0.078 (3)	0.154 (6)	0.072 (3)	−0.043 (4)	−0.012 (3)	0.001 (4)
O21	0.0365 (11)	0.0660 (19)	0.0551 (15)	−0.0086 (12)	−0.0030 (10)	−0.0042 (14)
O22	0.085 (2)	0.086 (3)	0.166 (4)	−0.023 (2)	−0.040 (2)	0.050 (3)
C21	0.065 (3)	0.073 (3)	0.061 (2)	−0.028 (2)	0.001 (2)	0.002 (2)
C22	0.080 (3)	0.112 (4)	0.086 (3)	−0.051 (3)	0.006 (2)	−0.007 (3)
O31	0.0348 (10)	0.0560 (15)	0.0539 (15)	0.0015 (11)	−0.0042 (10)	−0.0070 (13)
O32	0.0583 (15)	0.087 (2)	0.084 (2)	0.0141 (17)	−0.0288 (14)	−0.0235 (19)
C31	0.0339 (16)	0.060 (2)	0.050 (2)	−0.0046 (16)	−0.0002 (14)	−0.0076 (18)
C32	0.047 (2)	0.067 (3)	0.088 (3)	−0.005 (2)	−0.0027 (19)	−0.016 (3)
O41	0.0408 (11)	0.0665 (17)	0.0460 (14)	0.0019 (12)	0.0017 (10)	0.0056 (13)
O42	0.0414 (14)	0.110 (3)	0.083 (2)	0.0099 (17)	0.0072 (14)	0.001 (2)
C42	0.0407 (18)	0.055 (2)	0.060 (2)	−0.0067 (17)	0.0022 (16)	−0.0098 (19)
C43	0.079 (3)	0.067 (3)	0.070 (3)	−0.010 (2)	0.024 (2)	0.001 (2)
O51	0.0504 (14)	0.077 (2)	0.0671 (17)	−0.0206 (14)	−0.0168 (12)	0.0149 (16)
O52	0.075 (2)	0.095 (3)	0.158 (4)	−0.015 (2)	0.020 (2)	0.042 (3)
C51	0.063 (2)	0.062 (3)	0.061 (3)	−0.017 (2)	−0.0092 (19)	0.006 (2)
C52	0.057 (2)	0.068 (3)	0.080 (3)	−0.015 (2)	−0.005 (2)	−0.004 (3)
C53	0.063 (3)	0.106 (4)	0.091 (3)	−0.006 (3)	−0.020 (2)	0.000 (3)

### Geometric parameters (Å, °)

**Table:**

O1—C5	1.428 (4)	C12—H12A	0.9300
O1—C1	1.427 (4)	C13—C14	1.363 (12)
C1—O2	1.384 (4)	C13—H13A	0.9300
C1—C2	1.508 (5)	C14—C15	1.374 (10)
C1—H1A	0.9800	C14—H14A	0.9300
C2—O21	1.433 (4)	C15—H15A	0.9300
C2—C3	1.515 (5)	O21—C21	1.357 (6)
C2—H2A	0.9800	O22—C21	1.179 (5)
C3—O31	1.441 (4)	C21—C22	1.490 (6)
C3—C4	1.511 (4)	C22—H22A	0.9600
C3—H3A	0.9800	C22—H22B	0.9600
C4—O41	1.440 (4)	C22—H22C	0.9600
C4—C5	1.516 (5)	O31—C31	1.362 (4)
C4—H4A	0.9800	O32—C31	1.186 (4)
C5—C51	1.510 (5)	C31—C32	1.478 (6)
C5—H5A	0.9800	C32—H32A	0.9600
O2—C6	1.436 (5)	C32—H32B	0.9600
C6—C7	1.506 (5)	C32—H32C	0.9600
C6—H6A	0.9700	O41—C42	1.357 (4)
C6—H6B	0.9700	O42—C42	1.184 (5)
C7—N1	1.443 (4)	C42—C43	1.486 (6)
C7—H7A	0.9700	C43—H43A	0.9600
C7—H7B	0.9700	C43—H43B	0.9600
N1—C11	1.387 (6)	C43—H43C	0.9600
N1—C8	1.392 (6)	O51—C52	1.338 (5)
C8—O8	1.198 (6)	O51—C51	1.448 (5)
C8—C9	1.485 (6)	O52—C52	1.181 (6)
C9—C10	1.368 (7)	C51—H51A	0.9700
C9—C12	1.376 (7)	C51—H51B	0.9700
C10—C15	1.387 (7)	C52—C53	1.467 (7)
C10—C11	1.492 (7)	C53—H53D	0.9600
C11—O11	1.208 (6)	C53—H53A	0.9600
C12—C13	1.414 (10)	C53—H53B	0.9600
			
C5—O1—C1	112.5 (2)	C9—C12—C13	116.2 (7)
O2—C1—O1	107.1 (3)	C9—C12—H12A	121.9
O2—C1—C2	109.5 (3)	C13—C12—H12A	121.9
O1—C1—C2	107.6 (3)	C14—C13—C12	122.4 (7)
O2—C1—H1A	110.8	C14—C13—H13A	118.8
O1—C1—H1A	110.8	C12—C13—H13A	118.8
C2—C1—H1A	110.8	C13—C14—C15	120.4 (7)
O21—C2—C1	109.6 (3)	C13—C14—H14A	119.8
O21—C2—C3	108.5 (3)	C15—C14—H14A	119.8
C1—C2—C3	110.8 (3)	C14—C15—C10	117.8 (8)
O21—C2—H2A	109.3	C14—C15—H15A	121.1
C1—C2—H2A	109.3	C10—C15—H15A	121.1
C3—C2—H2A	109.3	C21—O21—C2	118.3 (3)
O31—C3—C4	108.2 (2)	O22—C21—O21	123.3 (4)
O31—C3—C2	107.3 (3)	O22—C21—C22	125.9 (5)
C4—C3—C2	110.6 (3)	O21—C21—C22	110.7 (4)
O31—C3—H3A	110.2	C21—C22—H22A	109.5
C4—C3—H3A	110.2	C21—C22—H22B	109.5
C2—C3—H3A	110.2	H22A—C22—H22B	109.5
O41—C4—C3	109.0 (2)	C21—C22—H22C	109.5
O41—C4—C5	107.2 (3)	H22A—C22—H22C	109.5
C3—C4—C5	111.6 (3)	H22B—C22—H22C	109.5
O41—C4—H4A	109.7	C31—O31—C3	118.9 (3)
C3—C4—H4A	109.7	O32—C31—O31	123.7 (4)
C5—C4—H4A	109.7	O32—C31—C32	126.5 (4)
O1—C5—C51	107.0 (3)	O31—C31—C32	109.9 (3)
O1—C5—C4	110.0 (3)	C31—C32—H32A	109.5
C51—C5—C4	113.1 (3)	C31—C32—H32B	109.5
O1—C5—H5A	108.9	H32A—C32—H32B	109.5
C51—C5—H5A	108.9	C31—C32—H32C	109.5
C4—C5—H5A	108.9	H32A—C32—H32C	109.5
C1—O2—C6	113.1 (3)	H32B—C32—H32C	109.5
O2—C6—C7	108.6 (3)	C42—O41—C4	117.8 (3)
O2—C6—H6A	110.0	O42—C42—O41	122.8 (4)
C7—C6—H6A	110.0	O42—C42—C43	125.6 (4)
O2—C6—H6B	110.0	O41—C42—C43	111.6 (3)
C7—C6—H6B	110.0	C42—C43—H43A	109.5
H6A—C6—H6B	108.4	C42—C43—H43B	109.5
N1—C7—C6	112.6 (3)	H43A—C43—H43B	109.5
N1—C7—H7A	109.1	C42—C43—H43C	109.5
C6—C7—H7A	109.1	H43A—C43—H43C	109.5
N1—C7—H7B	109.1	H43B—C43—H43C	109.5
C6—C7—H7B	109.1	C52—O51—C51	118.0 (3)
H7A—C7—H7B	107.8	O51—C51—C5	108.1 (3)
C11—N1—C8	111.9 (4)	O51—C51—H51A	110.1
C11—N1—C7	123.8 (4)	C5—C51—H51A	110.1
C8—N1—C7	124.3 (4)	O51—C51—H51B	110.1
O8—C8—N1	125.6 (4)	C5—C51—H51B	110.1
O8—C8—C9	128.5 (5)	H51A—C51—H51B	108.4
N1—C8—C9	105.9 (4)	O52—C52—O51	122.9 (5)
C10—C9—C12	121.2 (5)	O52—C52—C53	124.8 (4)
C10—C9—C8	108.1 (4)	O51—C52—C53	112.3 (4)
C12—C9—C8	130.7 (6)	C52—C53—H53D	109.5
C9—C10—C15	122.0 (5)	C52—C53—H53A	109.5
C9—C10—C11	108.5 (4)	H53D—C53—H53A	109.5
C15—C10—C11	129.5 (6)	C52—C53—H53B	109.5
O11—C11—N1	125.0 (4)	H53D—C53—H53B	109.5
O11—C11—C10	129.5 (5)	H53A—C53—H53B	109.5
N1—C11—C10	105.5 (5)		

### Hydrogen-bond geometry (Å, °)

**Table:**

*D*—H···*A*	*D*—H	H···*A*	*D*···*A*	*D*—H···*A*
C32—H32B···O42^i^	0.96	2.45	3.317 (5)	151
C43—H43C···O42^ii^	0.96	2.48	3.284 (5)	141

Symmetry codes: (i) −*x*, *y*+1/2, −*z*+1; (ii) −*x*, *y*−1/2, −*z*+1.
